# MicroRNA-128 Confers Anti-Endothelial Adhesion and Anti-Migration Properties to Counteract Highly Metastatic Cervical Cancer Cells’ Migration in a Parallel-Plate Flow Chamber

**DOI:** 10.3390/ijms22010215

**Published:** 2020-12-28

**Authors:** Pei-Chin Chuang, Chun-Wun Lu, Ching-Chin Tsai, Shun-Hung Tseng, Wen-Hong Su

**Affiliations:** 1 Department of Medical Research, Kaohsiung Chang Gung Memorial Hospital, Kaohsiung 833, Taiwan; pcjchuang@gmail.com (P.-C.C.); ryo0817@gmail.com (C.-W.L.); g30531@yahoo.com.tw (C.-C.T.); kkman110150@hotmail.com (S.-H.T.); 2 Stem Cell Research Core Laboratory, Department of Medical Research, Kaohsiung Chang Gung Memorial Hospital, Kaohsiung 833, Taiwan; 3 Department of Biotechnology, Kaohsiung Medical University, Kaohsiung 807, Taiwan

**Keywords:** *microRNA-128*, cervical cancer cells, cell adhesion, cell migration, parallel-plate flow chamber

## Abstract

Despite the distant metastasis of cervical cancer cells being a prominent cause of mortality, neither the metastasis capacity nor the in vitro conditions mimicking adhesion of cervical cancer cells to endothelial cells have been fully elucidated. Circulating metastatic cancer cells undergo transendothelial migration and invade normal organs in distant metastasis; however, the putative molecular mechanism remains largely uncertain. In this study, we describe the use of an in vitro parallel-plate flow chamber to simulate the dynamic circulation stress on cervical cancer cells and elucidate their vascular adhesion and metastasis. We isolate the viable and shear stress-resistant (SSR) cervical cancer cells for mechanistic studies. Remarkably, the identified SSR-HeLa and SSR-CaSki exhibited high in vitro adhesive and metastatic activities. Hence, a consistently suppressed *miR-128* level was revealed in SSR cell clones compared to those of parental wild-type (WT) cells. Overexpressed *miR-128* attenuated SSR-HeLa cells’ adherence to human umbilical cord vein endothelial cells (HUVECs); in contrast, suppressed *miR-128* efficiently augmented the static adhesion capacity in WT-HeLa and WT-CaSki cells. Hence, amplified *miR-128* modestly abolished in vitro SSR-augmented HeLa and CaSki cell movement, whereas reduced *miR-128* aggravated the migration speed in a time-lapse recording assay in WT groups. Consistently, the force expression of *miR-128* alleviated the SSR-enhanced HeLa and CaSki cell mobility in a wound healing assay. Notably, miR-128 mediated SSR-enhanced HeLa and CaSki cells’ adhesion and metastasis through suppressed *ITGA5*, *ITGB5*, *sLex*, *CEACAM-6*, *MMP9*, and *MMP23* transcript levels. Our data provide evidence suggesting that *miR-128* is a promising microRNA that prevented endothelial cells’ adhesion and transendothelial migration to contribute to the SSR-enhanced adhesion and metastasis progression under a parallel-plate flow chamber system. This indicates that the nucleoid-based *miR-128* strategy may be an attractive therapeutic strategy to eliminate tumor cells resistant to circulation shear flow, prevent vascular adhesion, and preclude subsequent transendothelial metastasis.

## 1. Introduction

Cervical cancer is one of the most prevalent oncological diseases in gynecologic medicine, with 570,100 newly diagnosed cases and 280,000 fatalities worldwide each year [1]. Surgery and radiation are common treatment options for patients with the early stages of cervical cancer [2]. Chemotherapy is often recommended for cases with the risk of postoperative recurrence, whereas combined regimes with chemotherapy and radiation are employed for those with late-stage disease [3,4]. Growing evidence has demonstrated that metastatic cervical cancer decreases the efficacy of radiotherapy or chemotherapy, and metastasis is the leading cause of death in cervical cancer patients [5,6]. However, the mechanisms underlying the distant metastasis of cervical cancer remain elusive.

Metastasis is one of the most important biological characteristics of malignant tumors. Cancer cells are carried toward other organs and subjected to invasion, seeding, and growth in distant normal tissues after being shed from primary tumors into the bloodstream. Among these processes, adhesion of circulating cancer cells onto the endothelial lining of blood vessels is an early, critical feature of distant metastasis that predicts cancer-associated mortality [7,8]. Consequently, survival cancer cells adhere to endothelial cells, transmigrate across the endothelium of blood vessels, and then metastasize to secondary sites [9,10]. These processes involve many cell surface adhesion molecules and various other cellular molecules. For example, several cancer cells’ surface-expressed integrins (such as αvβ3, β5 integrins) [11,12], ligand sialyl Lewis (sLex), and endothelial receptors (ICAM-1,VCAM-1) reportedly contribute to the adhesion of cancer cells to endothelia [13,14]. Circulating cancer cells rolling over the vascular walls are regulated by the engagement of endothelial cell-expressed E-selectin and P-selectin with tumor cell surface-expressed glycol proteins such as CD44, CD24 [9], sulfate-glycosaminoglycans (CS-GAGs) [15], and sialylated glycosphingolipids [16]. Although many previous reports have focused on the mechanisms underlying cancer cell local invasion for distant metastasis [17,18], the mechanistic events by which cervical cancer cells survive in the circulating stream in blood vessels and by which viable cervical cancer cells trigger vascular adhesion and later metastasis remain largely elusive. Moreover, an in vitro model to simulate the dynamic circulation stress of cervical cancer cells in vascular adhesion and metastasis is worth exploring. 

MicroRNAs (miRNAs) are endogenously produced from short noncoding RNAs of about 20–24 nucleotides that have been shown to influence numerous cancer-relevant processes [19]. *MicroRNA-128* (*miR-128*, which is on chromosome 2q21.3) has been shown to play an important role in the development of the nervous system and the maintenance of its normal physical functions [20]. Recently, Gao et al. have addressed the fact that *miR-128* can regulate proliferation, differentiation, and apoptosis via targeting Bmi-1, which is mainly related to the PI3K-AKT-mTOR signal pathway in neuroblastoma cells and ovarian cancer cells [21,22]. Zhu et al. demonstrated that ectopic expression of *miR-128* increases breast cancer cells’ apoptosis and DNA damage by directly targeting ABCC5 (multidrug resistance-associated protein5) expression when cooperating with doxorubicin in SK-3rd and MCF-7 [23]. This evidence supports the notion that *miR-128* is necessary for the regulation of cell apoptosis or tumor cell growth. Nevertheless, owing to the fact that most miRNAs are highly pleiotropic and differential in distinct cell types, the detailed function and regulation of *miR-128* involved in cervical cancer pathogenesis, such as the survival/resistance of cervical cancer in the blood circulation to facilitate later vascular adhesion or advanced metastasis, remain largely uncharacterized. 

A parallel-plate flow chamber, characterized by well-designed dynamic flow fluid, is a sophisticated device used to mimic in vivo physiological shear stress of 0.01–30 dynes/cm^2^ in various cell cultures [24]. Via an adjustable oscillatory pump, shear stresses over the immobilized substrate in the chamber are kinematically generated by various rates of flowing fluid. This system has been widely employed to evaluate the adhesion between peripheral blood leukocytes and vascular endothelial cells [25,26], for in vitro mimicking of hypertension and arthrosclerosis conditions [27], to evaluate the chemotaxis properties of cell cultures [28], and for drug delivery [29]. Lately, growing evidence has suggested that a parallel-plate flow chamber is an ideal in vitro simulation model to detect the movement of cell cultures exposed to blood circulation [30,31]. We have previously successfully invented several unique flow chamber systems for layering collagen or vessel tissues to visualize intracellular events including adhesion, transendothelial migration, and the extravasation of leukocytes while mimicking in vivo dynamic shear stress [32,33,34]. In this study, we employed a parallel-plate flow chamber to isolate cervical cancer cells with high adhesion capacity with endothelial cells. We hypothesized that a parallel-plate flow chamber system would be an ideal strategy to simulate in vivo dynamic circulation conditions for selection of shear stress-resistant (SSR) cervical cancer cells. We also aimed to elucidate whether the highly shear stress-resistant cervical cancer cells isolated by the flow chamber system have a high metastasis capacity. Furthermore, we investigated the novel roles of *miR-128* in the regulation of resistance to shear stress, adhesion to endothelial cells, and the migrative properties of circulating cervical cancer cells, as well as the molecular mechanisms. The modulation of *miR-128* signaling may be a unique way to prevent cervical cancer cells’ vascular adhesion and subsequent distal migration.

## 2. Results

### 2.1. Employing a Parallel-Plate Flow Chamber System for the Selection of Wild-Type and Shear Stress-Resistant Cervi-Cal Cancer Cells’ Clones, and a Decreased Expression Level of miR-128 Was Observed in the SSR Group Compared to the WT Group 

**Figure 1:**
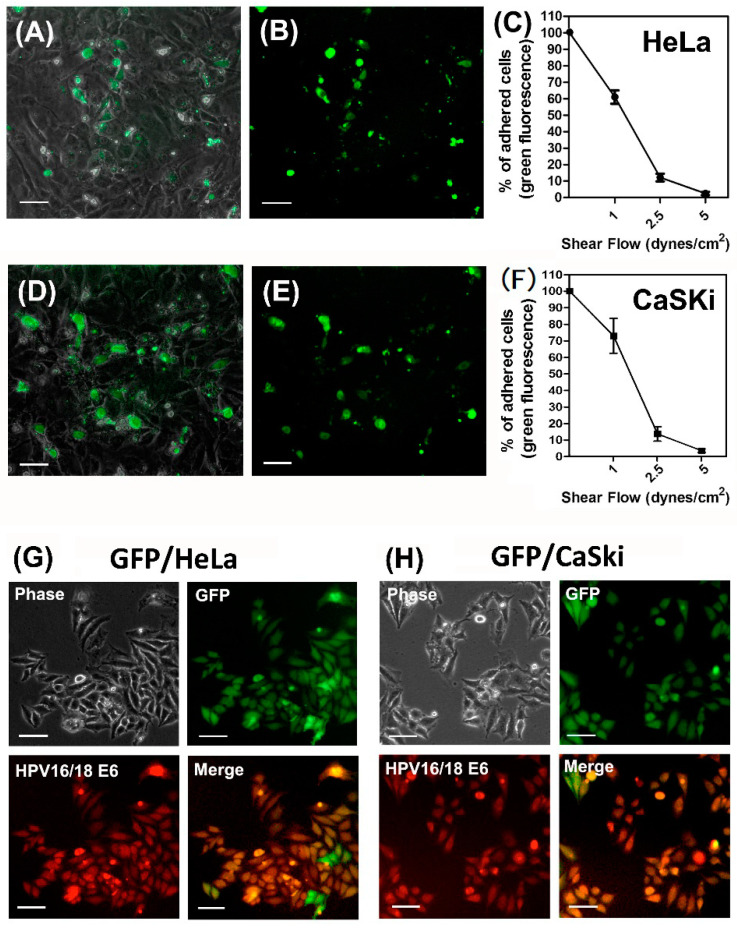
Establishment of a parallel-plate flow chamber system to simulate the in vivo dynamic flowing conditions for a selection of wild-type (WT) and shear stress-resistant (SSR) cervical cancer cell clones, and decreased expression of *microRNA-128* (*miR-128*) in the SSR group compared to the WT group. A parallel-plate flow chamber was assembled with a glass slide seeded on a monolayer of human umbilical cord vein endothelial cells (HUVECs) and suffused for 24 h with a medium for HUVECs; the various shear flow forces were generated and controlled using a peristaltic pump. (**A**) Representative microscopic images show that the GFP-expressed HeLa cells remained on the HUVEC layer after being exposed to 2.5 dynes/cm^2^ shear forces for 10 min. (**B**) Viable GFP-expressed SSR-HeLa cells shown in green fluorescence. Scale bar, 100 μm. (**C**) Summary of the ratio of adhered cells on the HUVEC layer after exposure to various shear flow forces. Consistent results are shown in (**D**,**E**); the viable GFP-expressed CaSki cells that adhered on the HUVEC layer were resistant to 2.5 dynes/cm^2^ shear flow. The ratio of adhered CaSki cells on the endothelial cells was counted and summarized in (**F**). (**G**) Representative figures show the phase contrast (left upper panel) of GFP-expressed WT-HeLa cell populations indicated by a FACSAria™ III cell sorter. Isolated GFP-expressed HeLa cell populations are shown in green fluorescence (right upper panel) and HPV 16/18 E6-stained HeLa are shown in red fluorescence (left lower panel). The merged photographs (shown in yellow fluorescence; right lower panel) indicate that GFP-expressed HeLa cells co-expressed HPV 16/18 E6, a marker only shown on HeLa and CaSki cervical cells instead of in HUVECs. Similar results were found in isolated GFP-expressed SSR-CaSki cells, as shown in (**H**). Scale bar, 100 μm. This implies that our isolated GFP-expressed HeLa and CaSki cells clones were free of contamination from HUVECs.

**Figure 2:**
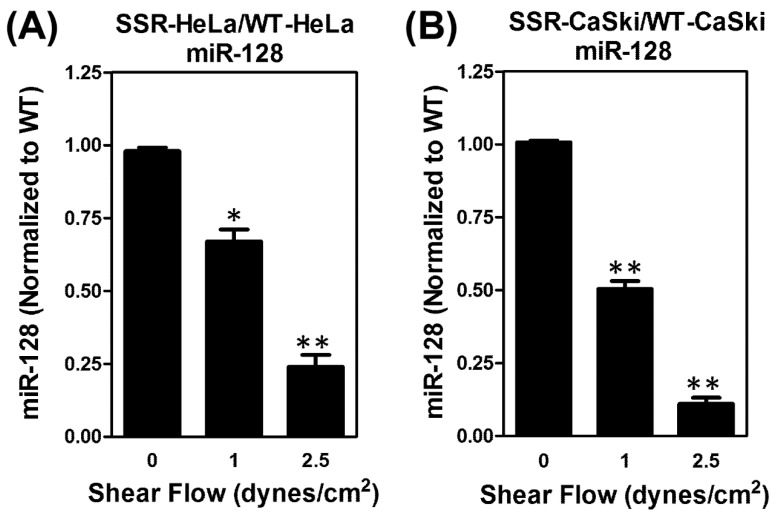
Suppression of *miR-128* level in isolated WT and SSR cervical cancer cells as measured by parallel-plate flow chamber system. Isolated GFP-expressed WT or SSR cell populations (resistant to 0, 1, or 2.5 dynes/cm^2^ shear flow) were sorted by cell sorter FACSAria™ III (BD Bioscience) to separate out HUVEC endothelial cells and we then extracted the total microRNA and expression levels of *miR-128* through a quantitative RT-PCR analysis. Notably, consistent downregulation of *miR-128* was shown for (**A**) SSR-HeLa and (**B**) SSR-CaSki cells clones in a shear stress-dependent manner. The data show the mean and standard deviation (SD) of three independent experiments using different batches of cells. * *P* < 0.05; ** *P* < 0.01 for the shear flow of 1 or 2.5 dynes/cm^2^ group vs. control group.

Our group and others have previously published on the use of a parallel-plate flow chamber system that contains a cover glass slide inoculated with a monolayer of human umbilical vessel endothelial cells (ECs); we also employed oscillatory peristaltic pumps to generate various shear forces through the chamber [32,33]. For this current study, a schematic diagram of the parallel-plate flow chamber and working flowchart was shown in Figure S1. In brief, cervical cancer cells were perfused through the flow chamber to interact with ECs under various shear forces. The viable cervical cancer cells that adhered onto ECs were isolated as shear stress-resistant (cell clones. For wild-type (WT) control cell clones, the parental cells perfused in the flow chamber without shear flow were harvested. To visualize adherent cervical cancer cells, all of the cervical cancer cells (HeLa and CaSki) were transected with green fluorescent protein (GFP), as described in Section 4.6. Fluorescence microscopic observation showed that viable GFP-HeLa cells resistant to shear stress were able to adhere onto ECs (Figure 1A,B). The number of HeLa cells adhering to ECs decreased with the shear forces (Figure 1C). A similar procedure was performed to count the viable GFP-CaSki cells resistant to shear stress that remained on ECs (Figure 1D,E), and we also observed that the number of CaSki cells adhering to ECs decreased with the shear force (Figure 1F). Shear stress-resistant HeLa cells were defined as previously described [8]. Therefore, we collected shear stress-resistant HeLa cell clones (which were viable and resistant to 2.5 dyn/cm^2^ of shear stress; here, we defined these as SSR clones) and increased the number of isolated in vitro clones for further mechanistic study. The isolated GFP-expressed WT- and SSR-HeLa clones were indicated by green fluorescence (Figure 1B,E). We then further co-stained a selective human papillomavirus 16/18 E6 oncoprotein (red fluorescence) with sorter-isolated GFP-expressed WT-HeLa cells (Figure 1G) and WT-CaSki cells (Figure 1H) to confirm the identities of cervical cancer cells, since the HeLa and CaSki cells have been well characterized the HPV-18/E6 and HPV-16/E6 integrated cervical cancer cell lines [34]. We then evaluated the expression levels of the *miR-128* in isolated GFP-expressed SSR (resistant to 0, 1, or 2.5 dynes/cm^2^ shear flow) or parental WT cervical cancer cell clones (Figure 2). Notably, we found that the *miR-128* level was consistently suppressed in SSR-HeLa and SSR-CaSki cell clones compared to those of the parental WT group through quantitative RT-PCR analysis. These data provide evidence that the parallel-plate flow chamber system is an ideal strategy to simulate in vivo dynamic circulation conditions and effectively select viable and circulating HeLa or CaSki cervical cancer cell clones that are resistant to shear flow and adhere onto ECs. In addition, we found a unique and consistent feature, a suppressed *miR-128* level in a shear stress-dependent manner, in SSR-HeLa and SSR-CaSki cervical cancer cell clones. 

### 2.2. SSR Cervical Cancer Cells Had High Adhesive Capacities and Ectopic Expression of an miR-128 mimic Attenuated SSR Cervical Cancer Cells’ Adherence to Endothelial Cells In Vitro 

**Figure 3:**
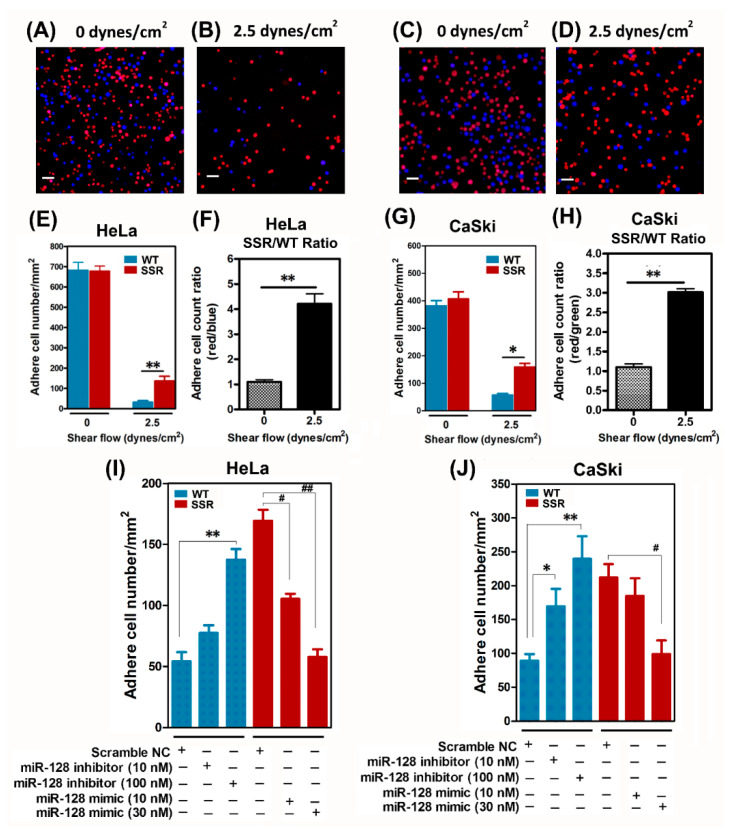
Increased static adhesion capacities in SSR-HeLa and SSR-CaSki cervical cancer cells measured by a parallel-plate flow chamber system. Overexpression of miR-128 reduced the SSR-enhanced cervical cancer cells’ adhesion to endothelial cells. The adhesion capacity of the SSR and WT clones was measured by a static adhesion assay, as described in Section 4.7. In brief, mixtures containing 1 × 10^5^ Orange Red CMRA prelabeled SSR cells and 1 × 10^5^ Blue CMF2HC prelabeled WT cells were gently infused into the flow chamber and kept there for 12 min to let the cervical cancer cells settle onto the human umbilical cord vein endothelial cell (HUVEC) layer. This was followed by perfusion with 0 or 2.5 dyn/cm^2^ shear flow for 10 min. HeLa cells adhered to HUVECs after (**A**) 0 dyn/cm^2^ or (**B**) 2.5 dyn/cm^2^ shear flow for 10 min. Similar, CaSki cells was shown to adhere on HUVECs after (**C**) 0 dyn/cm^2^ or (**D**) 2.5 dyn/cm^2^ shear flow for 10 min. Scale bar, 100 μm. (**E**,**F**) In HeLa cells, the SSR cells adhered on the HUVECs layer (red fluorescence) and WT cells (blue fluorescence; without exposure to shear stress) and the cell ratio of SSR/WT was nearly 1:1. Increased SSR cells’ adherence onto the ECs compared to WT cells was seen after exposure to 2.5 dynes/cm^2^ shear stress for 10 min. (**F**) Data are expressed as the number of cells positive for red fluorescence (SSR)/the number of cell positive for blue fluorescence (WT). A similar phenomenon was observed in CaSki cell clones (**G**,**H**). (**I**) HeLa or (**J**) CaSki cells were transfected with synthetic *miR-128* inhibitor oligonucleotides, miR-128 mimic oligonucleotides, or scramble negative control oligonucleotides for 48 h and then we performed the static adhesion assay. All the experiments were repeated three times and at least six fields were counted per experiment. Each dataset was reported as the mean ± SD; * *P* < 0.05, ** *P* < 0.01 for the WT + *miR-128* inhibitor group vs. WT group; # *P* < 0.05, ## *P* < 0.01 for the SSR + *miR-128* mimic group vs. SSR group.

Next, we verified the adhesive capacities of SSR clones and WT clones using a static adhesion assay protocol [35], as described in Section 4.7. Briefly, mixtures containing 1 × 10^5^ prelabeled CMRA SSR cells and 1 × 10^5^ prelabeled CMF2HC WT cells were perfused through a flow chamber and the number of SSR and WT cells that adhered to the EC layer was counted under a fluorescence microscope. The SSR cells on the EC layer displayed a red fluorescent reaction and WT cells exhibited blue fluorescence; the cell ratio of SSR and WT clones was nearly 1:1 before the exposure to shear stress (see Figure 3A for HeLa; Figure 3C for CaSki). More SSR cells than WT cells adhered onto the EC layers after exposure to 2.5 dynes/cm^2^ shear stress for 10 min, suggesting the higher adhesion capacity of the SSR group (Figure 3B for HeLa; Figure 3D for CaSki). Image analysis using the cell count Wimasis GmbH image analysis software confirmed that the SSR group had a higher number of cells adhering onto the EC layer than the WT group after exposure to 2.5 dynes/cm^2^ shear stress (Figure 3E for HeLa; Figure 3G for CaSki). The SSR group had higher adhesion capacity compared to the WT group (ratio of adherence in SSR/WT cell count; Figure 3F for HeLa and Figure 3H for CaSki). Furthermore, we wanted to explore whether the loss/gain of function of *miR-128* affected the capacity of SSR-enhanced cervical cancer cells to adhere onto ECs. The validation of *miRNA-128* level in WT or SSR cervical cancer cells after transfection of scramble negative control, *miR-128 inhibitor*, or *miR-128 mimic* oligonucleotides was shown in Figure S2. As shown in Figure 3I, the administration of WT-HeLa with *miR-128 inhibitor* dose-dependently enhanced the adhesive abilities compared to the scramble negative control (NC) group; in contrast, transfection of *miR-128 mimic* efficiently attenuated the adhesive capacities of SSR-HeLa selected under 2.5 dynes/cm^2^ shear stress. Similar predominance was shown in CaSki cell clones, where forced expression of *miR-128* markedly reduced the SSR-CaSki cell adhesive properties, whereas a decrease in *miR-128* greatly increased the adhesive capacity of WT-CaSki to ECs (Figure 3J). Our data showed that *miR-128* had an in vitro anti-adhesion property sufficient to eradicate the SSR-HeLa- and SSR-CaSki-augmented cell adherence to endothelial cells. 

### 2.3. SSR Cervical Cancer Cells Had Higher Migrative Capacity Compared to Parental WT Clones, and Exogenous Administration of miR-128 Mimic Attenuated SSR-Enhanced Cell Migration and Wound Healing In Vitro 

**Figure 4:**
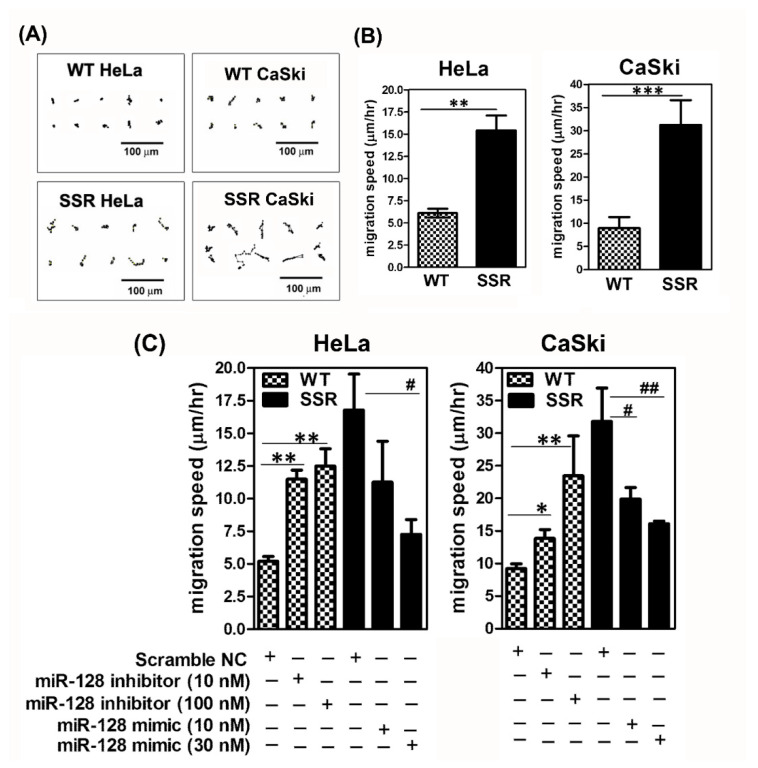
Shear stress-resistant CaSki and HeLa cells exhibited enhanced cell mobility in vitro and forced overexpressed *miR-128* suppressed SSR-augmented cervical cancer cell migration. (**A**) Representative cell migration trace from a time-lapse recording of cell migration over 8 h. The result was representative of various independent clones of WT and SSR clones of HeLa and CaSki cells. Scale bar, 100 μm. (**B**) Summary of migration speed by time-lapse recording assay of the migration of HeLa and CaSki cells over 8 h. Data are shown as mean ± SD. All the experiments were repeated three times and calculated from at least 20 cells per field for individual samples; eight fields from each experiment were counted from at least two repeated experiments. ** *P* < 0.01, *** *P* < 0.001 for the SSR group vs. WT group. (**C**) HeLa or CaSki cells were transfected with *miR-128* inhibitor oligonucleotides, miR-128 mimic oligonucleotides, or scramble negative control oligonucleotides for 48 h; then we performed the time-lapse recording assay. Each dataset was reported as the mean ± SD; * *P* < 0.05, ** *P* < 0.01 for the WT + *miR-128* inhibitor group vs. WT + scramble NC group and SSR + *miR-128* mimic group vs. SSR + scramble NC group. # *P* < 0.05, ## *P* < 0.01 for the SSR + miR-128 mimic group vs. SSR + scramble NC group.

**Figure 5:**
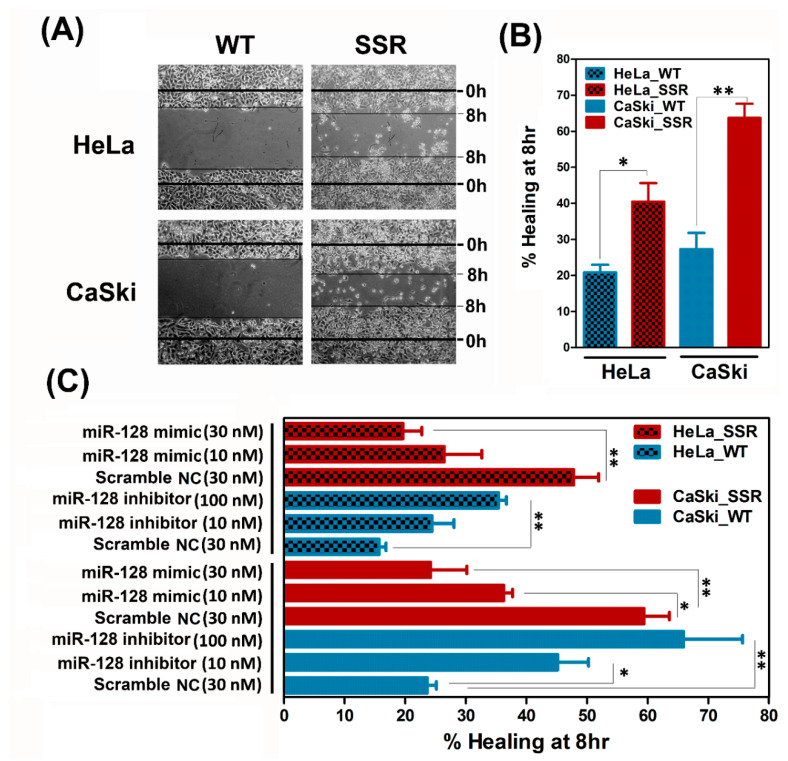
Isolated shear stress-resistant CaSki and HeLa cells augmented cell migration by the wound healing assay and overexpression of *miR-128* suppressed the SSR-enhanced cervical cancer cell healing property. (**A**) Representative phase-contrast micrographs depicting scratched monolayers of cultured WT- and SSR-HeLa and CaSki cells at 0 and 8 h. Scale bar, 100 μm. (**B**) Summary of wound healing 8 h after scratching. * *P* < 0.05; ** *P* < 0.01 for the SSR group vs. WT group. (**C**) Administration of *miR-128* inhibitor significantly increased the WT-HeLa and WT-CaSki cells’ wound healing rate compared to the scramble NC control group. Overexpression of *miR-128* successfully diminished the healing rate of SSR-HeLa and SSR-CaSki cells compared to those in the scramble NC group. Each dataset was reported as the mean ± SD. All the experiments were repeated three times and at least six fields were counted per experiment. * *P* < 0.05, ** *P* < 0.01 for the WT + *miR-128* inhibitor group vs. WT + scramble NC group and SSR + *miR-128* mimic group vs. SSR + scramble NC group.

We furthermore explored the impact of *miR-128* on the modulation of the SSR-enhanced in vitro migration of cervical cancer cells. A time-lapse recording of cell migration and a wound healing assay were performed on the parental WT and SSR cervical cell clones, as described in Section 4.8 and Section 4.9, respectively. As shown in Figure 4A, for SSR-HeLa, SSR-CaSki, and their parental WT clones, time-lapse recordings of movement were made hourly to trace the paths of migrating cells. Quantitative migration speed results showed that, consistently, both the shear stress-resistant HeLa and CaSki cells migrated faster than their wild-type counterparts (Figure 4B). Later, loss/gain of function of *miR-128* was performed to examine the roles of *miR-128* in SSR-enhanced cervical cancer adhesion. It is noteworthy that the forced introduction of *miR-128 mimic* into SSR-HeLa or SSR-CaSki cells substantially reduced the migration speed, whereas exogenous administration of *miR-128* inhibitor increased the SSR-HeLa and SSR-CaSki cell migrating capacity (Figure 4C). To further confirm that shear stress resistance was associated with augmented cell migration, the wound healing rate of SSR-HeLa, SSR-CaSki cells, and their parental WT control group was analyzed. As shown in Figure 5A,B, SSR-HeLa and SSR-CaSki cells showed a significantly amplified healing rate compared to wild-type cells. Notably, overexpression of *miR-128* effectively suppressed the healing rate of SSR-HeLa and SSR-CaSki cells compared to those in the scramble NC group (Figure 5C). In contrast, the reduced *miR-128* level obtained by the administration of *miR-128 inhibitor* apparently promoted the wound healing rate in both the WT-HeLa and WT-CaSki groups compared to the NC control group. Our data indicate that *miR-128* is a promising antimigration microRNA in vitro and may play a critical role in the regulation of cell mobility in SSR cervical cancer cells. 

### 2.4. MicroRNA-128 Exhibited Anti-Adhesion and Antimigration Properties to Alleviate Various SSR-HeLa- or CaSki-Augmented Critical Factors (e.g., Cell Adhesion, Extracellular Matrix Degradation, and Cell Migration) In Vitro 

**Figure 6:**
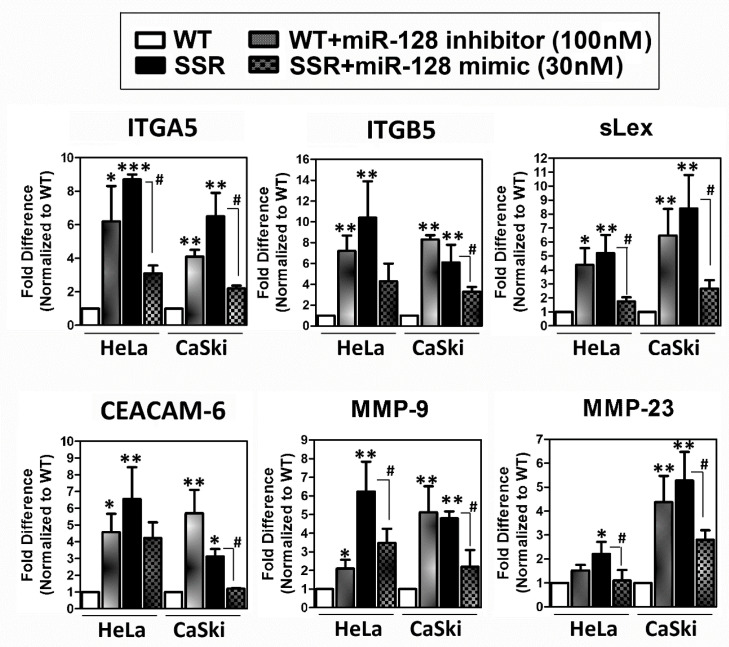
Effects of exogenous introduction of *miR-128* on the expression of bioinformatics-predicted *miR-128* targeted genes related to cell adhesion or cell invasion/metastasis. WT and SSR cells were transfected as described in Section 4.4 and then the cell extracts were collected for analysis of the transcription levels of various *miR-128* targeted genes using quantitative PCR. For the transcription levels determined using messenger RNA (mRNA) extracted from WT cells treated with *miR-128 inhibitor* or SSR cells treated with *miR-128 mimic*, the values were first normalized to those of the *18S ribosomal RNA* (abbreviated *18S rRNA*) internal control and then to the value obtained using mRNAs extracted from the WT scramble NC control. Data are given as mean ± SD; * *P* < 0.05, ** *P* < 0.01, *** *P* < 0.001 for the WT + *miR-128 inhibitor* group vs. WT + scramble NC group; ^#^ *P* < 0.05 for the SSR + *miR-128 mimic* group vs. SSR + scramble NC group.

We tried to clarify the molecular mechanisms responsible for the *miR-128*-mediated abrogation of the SSR-enhanced adhesive capacity of HeLa or CaSki cells, which may subsequently facilitate tumor cells’ transendothelial migration. To illustrate the impact of *miR-128* on the progression, we used bioinformatics (TargetScan Human: http://www.targetscan.org) to predict *miR-128* target genes and validated their expression levels using a quantitative RT-PCR assay. A bioinformatics analysis showed numerous *miR-128* predicted target genes of cell adhesion molecules such as *α5-integrin (ITGA5)*, *integrin β5 (ITGB5)*, *sialyl-Lewis X* (sLex; also called *CD15s* or *SSEA-1*), *focal adhesion kinase (FAK)*, and *carcinoembryonic antigen cell adhesion molecule 6* (*CEACAM6*). A TargetScan analysis also indicated that *miR-128* may directly target *matrix metallopeptidase (MMP)-9*, *MMP-23*, *H-Ras*, and *ROCK*, which manage the extracellular matrix degradation or cell invasion/metastasis. According to the results of in vitro adhesion and migration functional assays (Figure 3, Figure 4 and Figure 5), we picked the most effective dosage of the *miR-128* inhibitor (100 nM) or miR-*128 mimic* (30 nM) to perform experiments on the expression of cell adhesion or cell invasion/metastasis molecules. After validation by quantitative RT-PCR, we discovered that diminished *miR-128* in WT-HeLa or WT-CaSki cells markedly increased the expression levels of *ITGA5*, *ITGB5*, *sLex*, *CEACAM-6*, *MMP9*, and *MMP23* in WT-HeLa and WT-CaSki cervical cancer cell clones (Figure 6). In contrast, overexpression of *miR-128 mimic* significantly decreased those expression levels in both SSR-HeLa and SSR-CaSki cervical cancer cell clones selected from a parallel-plate flow chamber system (Figure 6). To further evaluated whether the application of *miR-128* oligonucleotides may alter the cervical cancer cell adhesions capacity via the *ITGA5* and *ITGB5*. We additionally performed an induvial experiment to evaluate the effects of knockdown of *ITGA5* or *ITGB5* on the static adhesion capacities in human cervical cancer cells, then elucidate the impact of *miR-128* on *ITGA5* or *ITGB5* mediated on the adhesive ability, and the data was shown in Figure S3. This data supported that *miR-128* exhibited anti-adhesion property to decline the SSR cervical cancer cell-enhanced adhesions capacity may via diminishing the ITGA5 or ITGB5 levels. In sum, our data indicate that *miR-128* blocks endothelial adhesion and the migration of SSR cervical cancer cells.

## 3. Discussion

Cervical cancer is one the most prevalent cancers in women worldwide. Distant metastasis is a leading cause of mortality. The mechanism underlying cervical cancer’s metastasis is still largely elusive. Circulating metastatic cancer cells going through transendothelial migration and invading normal organs is a feature of distant metastasis. We have previously reported on the invention of unique flow chamber systems [32,33,34]. In this study, we established an innovative parallel-plate flow chamber to simulate metastatic cancer cells’ survival in dynamic circulation and found that the number of viable cervical cancer cells adhering to the EC layer in the flow chamber decreased with shear stress. We then utilized this device to successfully isolate several viable shear stress-resistant cervical cancer cell clones (SSR-HeLa and SSR-CaSki) that adhere to endothelial cells under 2.5 dynes/cm^2^ dynamic shear stress. Notably, the identified SSR-HeLa and SSR-CaSki cells exhibited high levels of migratory and adhesion activities in vitro. We then discovered a unique feature: the *miR-128* level was consistently suppressed in SSR-HeLa and SSR-CaSki cells compared to in parental wild-type control cells. Exogenously introduced *miR-128* mimic significantly attenuated SSR-HeLa cells’ adherence to ECs; in contrast, suppression by *miR-128* inhibitor efficiently augmented the static adhesion capacity in WT-HeLa cells. Additionally, increased *miR-128* reduced in vitro SSR-augmented cervical cell movement, whereas reduced *miR-128* aggravated the parental WT control group cells’ migration speed in a time-lapse recording assay. Comparably, exogenous administration of *miR-128* mimic effectively alleviated the SSR-enhanced cell migrating mobility in a wound healing assay. Furthermore, forced introduction of *miR-128* mimic markedly diminished several *miR-128* targeted genes that control cell adhesion, extracellular matrix degradation, or cell migration/invasion, including *α5-integrin (ITGA5), integrin α5 (ITGB5)*, *sialyl-Lewis X (sLex)*, *carcinoembryonic antigen cell adhesion molecule 6 (CEACAM6)*, *metallopeptidase (MMP)-9,* and *MMP-23*. Collectively, our data show that the parallel-plate flow chamber is an ideal strategy for isolating circulating cervical cancer cells with high adhesive capacity to ECs and high migrative potential. We also identified the impacts of *miR-128*, which may advance the interruption of circulating cervical cancer cells’ adherence to the peripheral vascular endothelium and then block subsequent cell migration. In sum, our data sheds light on how the modulation of *miR-128* signaling might diminish the vascular adhesion of circulating cervical cancer cells and prevent cell migration.

Metastasis is one of the most important biological characteristics of malignant tumors. Certain cancer cells in primary tumors triggered by undefined mechanisms become aggressive cells that carry out metastasis. Metastasis is characterized by the movement of cancer cells away from the original tumor, their migration into the blood circulation or lymphatic system, and invasion of distant tissues and subsequent propagation [36]. Depending on the intrinsic properties of cancer cells, at least five events take place in the pathogenesis of distant metastasis [37,38], as outlined below: (1) local invasion: cancer cells are found to degrade the extracellular matrix (ECM) of the tumor and then escape from the primary tumor. (2) Intravasation: cancer cells are then vascularly transported in the hostile hemodynamic environment, rolling and adhering to endothelial cells in the peripheral vessel walls and invading the endothelium of blood vessels. (3) Survival in the circulation: few cancer cells survive in rigorous vasculature transportation conditions. (4) Arrest at a distant site: viable cancer cells transported through the bloodstream become resident in the environment of certain tissues or organs. (5) Transendothelial migration and extravasation: cancer cells transmigrate across the endothelium of blood vessels and extravasate into the parenchyma of targeted tissues. Then the cancer cells enter the connective tissue to form new metastatic lesions and gradually proliferate and develop to detectable neoplasm. Among these, very few cancer cells (less than 0.01% of tumor cells) reportedly penetrate into the circulation and, eventually, neoplasm development [39,40]. The number of circulating cancer cells in patients reportedly correlates with survival and the successful rate of modality [39,41]. For example, patients with metastasized breast cancer reportedly have a low survival rate as their peripheral blood contains five circulating tumor cells/7.5 mL [39,41]. These findings imply that surviving circulating metastatic cancer cells going through transendothelial migration and invading normal organs is a critical feature to initiate distant metastasis, and a feasible in vitro condition mimicking the adhesion of cervical cancer cells to endothelial cells is needed. Fortunately, the literature and our previous publications have demonstrated that a parallel-plate flow chamber is an ideal system to investigate the metastasis of various peripheral blood cells or cancer cells [32,33,34]. A parallel-plate flow chamber system is easily assembled and operated and allows scientists to integrate real-time microscopic videoing or imaging to simultaneously visualize the morphology and behavior of cell cultures exposed to dynamic shear stress. The system has been broadly employed to assess adhesion between peripheral blood leukocytes and vascular endothelial cells [25,26] and in vitro mimicking of hypertension and arthrosclerosis conditions [27]. This custom-designed system has been employed to analyze the interaction between endothelial cells and smooth muscle actin co-culture transwell modules [42]. Notably, a flow chamber system has recently been employed to elucidate the adhesion capacity of tumor cells onto vascular endothelium exposed to defined laminar flow conditions [43]. The parallel-plate flow chamber strategy has also been used for delineating the bone metastasis of tumor cells [44] and interaction between circulating tumor cells and endothelial cells [45], as well as the signaling transduction underlying the brain metastasis of prostate tumor cells DU-145 [46,47]. However, surprisingly, there is little information regarding the selection of a stable shear stress-resistant clone via a flow chamber system and the maintenance of clones for further study. Thus, we established a parallel-plate flow chamber to simulate metastatic cervical cancer cells that survive in dynamic circulation. We then successfully demonstrated that this device was able to select the viable shear stress-resistant cervical cancer cell clones (SSR-HeLa and SSR-CaSki) that adhere to ECs and expand these stable SSR clones in vitro for an extensive mechanistic study.

However, the cellular and molecular mechanisms underlying the adhesion and migration of shear stress-resistant cervical cancer cells remain uncertain. In this study, we demonstrated that the novel roles of *miR-128* may participate in the regulation of shear stress-resistant enhanced cervical cancer cells’ adhesion and migration, as well as further elucidating the potential mechanisms. MicroRNAs are endogenously produced, short, noncoding RNAs and have emerged as key regulators that contribute to various cancer-relevant processes [19]. More recently, numerous miRNAs have been documented to be dysregulated in various cancer cells and to regulate cancer adhesion and metastasis. Ling et al. have reported that *miR-145* inhibits lung cancer cell metastasis by targeting the Oct4-mediated Wnt/β catenin signaling pathway [48]. *MicroRNA-362-5p* promotes tumor growth and metastasis by targeting a cytoskeleton-associated protein, Cylindromatosis (CYLD), in hepatocellular carcinoma [49]. It has been reported that *miR-337-3p* attenuates tumor necrosis factor-related apoptosis-inducing ligand cytotoxicity in pancreatic ductal adenocarcinoma cells [50]. *MicroRNA-100* has been found to function as a tumor suppressor by inhibiting Lgr5 expression in colon cancer cells [51]. *MicroRNA-29a* eliminates lung adenocarcinoma cells’ growth, migration, and invasion by targeting carcinoembryonic antigen-related cell adhesion molecule 6 (CEACAM6) [52]. Kiss et al. reported a significant correlation between the expression changes of *miR-99b* and *miR-221* and the number of tissue eosinophils in the colonic mucosa of eosinophilic colitis patients, which may strongly correlate with the inflammation of tissue eosinophilia [53]. *MicroRNA-8* induces robust motor axon targeting by coordinated regulation of cell adhesion molecules (CAMs) during synapse development [54]. Suarez et al. have also demonstrated that the overexpression of some specific miRNAs, such as *miR-31* and *miR-17-3p*, in neutrophil, which subsequently inhibits certain adhesion molecules like E-selectin and intercellular adhesionmolecule-1 (ICAM-1) on cultured endothelial cells, results in the inhibition of neutrophil adhesion to cultured endothelial cells [55]. These studies indicate that miRNA may be an attractive therapeutic strategy to deal with tumor adhesion and metastatic progression. Hence, *miR-128* is reported to regulate proliferation, differentiation, and apoptosis in various cancers [21,22,23]. A recent study has also demonstrated that ectopic overexpression of *miR-128* in the neuroblastoma cell line SH-SY5Y effectively downregulates glioblastoma cell invasion by directly targeting an extracellular matrix glycoprotein, Reelin, and a microtubule-associated protein, DCX [56], which indicates the anti-cell invasion potency of *miR-128*. These findings are partially in line with our data. Notably, we revealed that the expression level of *miR-128* was steadily diminished through shear stress perfusion and isolated SSR-HeLa and SSR-CaSki cell clones, which exhibited consistently lower expression levels of *miR-128* than parental WT control clones (Figure 2). Reinforced overexpression of *miR-128* markedly attenuated SSR-HeLa or SSR-CaSki cells with enhanced in vitro static adhesive property (Figure 3I,J), and reintroduced *miR-128* reduced in vitro SSR-augmented cervical cell motility (Figure 4C). Our data also provide the first evidence that *miR-128* is an imperative anti-endothelial cell adhesion and antimigration microRNA and participates in the resistance to the shear stress that leads to reinforced cervical cancer cell adhesion and migration in a parallel-plate flow chamber system. 

Numerous studies have reported on the potential cellular mechanisms responsible for the endothelial adhesion of tumor cells and transendothelial migration. CD44 is reported to be essential for prostate cancer and breast cancer cell adhesion and transendothelial migration [9], as well as for enhanced cancer cell adhesion to bone marrow endothelial cells [57,58]. Cadherins contribute to the rolling and adhesion of breast carcinoma cells [59] and melanoma cells [60] onto the endothelium. Integrins (such as αvβ3 and β5 integrins) [11,12], ligand sialyl Lewis [13,14], and endothelial receptors (ICAM-1,VCAM-1) [3,14] are also widely reported to contribute to the adhesion of cancer cells to endothelia in and expressed on several cancer cells’ surface. However, how *miR-128* integrates into shear stress-resistant enhanced cervical cancer cells’ adhesion and metastasis is uncertain and needs further investigation. Bioinformatics analysis showed that several *miR-128*-predicted target genes involve cell adhesion, extracellular matrix degradation, or cell invasion/metastasis. We further validated that the overexpression of *miR-128* markedly decreased *ITGA5, ITGB5, sLex, CEACAM-6, MMP-9*, and *MMP-23* in SSR-HeLa and SSR-CaSki cervical cancer cell clones (Figure 6). These data suggest that *miR-128* indeed carries out crucial activities against endothelial adhesion and transendothelial migration. To our knowledge, our findings regarding the molecular mechanisms of *miR-128*’s participation in shear stress resistance-led cervical cancer cells’ adhesion and migration via a series of potent *miR-128* targeted molecules in a parallel-plate flow chamber system is novel. 

In this study, we established an in vitro parallel-plate flow chamber module to simulate the dynamic circulation stress and observed circulating cervical cancer cells’ vascular adhesion and cell migration. We isolated and expanded viable shear stress-resistant cervical cancer cells for mechanistic studies. Our data suggest that *miR-128* is a promising microRNA with anti-endothelial cell adhesion and antimigration features that hinder SSR cervical cancer cells’ adhesion and migration in a parallel-plate flow chamber system. *MicroRNA-128* alleviated SSR-enhanced HeLa and CaSki cells’ adhesion and metastasis through suppressing numerous molecules, including *ITGA5, ITGA5, sLex, CEACAM-6, MMP9*, and *MMP23*. The evidence indicates that the nucleoid-based *miR-128* strategy may be an attractive therapeutic strategy against tumor cells resistant to the shear flow of blood circulation and to prevent vascular adhesion and subsequent cervical cancer cell migration.

## 4. Materials and Methods

### 4.1. Cultivation of Cervical Cancer Cells

Human cervical carcinoma cell cultures HeLa and CaSki are widely employed for studies of cervical cancer cell behavior [61,62]. We purchased HeLa and CaSki cells from the American Type Culture Collection (ATCC, Manassas, VA, USA) and incubated them in a basal medium containing Dulbecco’s Modified Eagle’s tissue culture medium (DMEM; for HeLa: Sigma-Aldrich, St. Louis, MO, USA) or Roswell Park Memorial Institute Medium (RPMI 1640; for CaSki: Thermo Fisher Scientific, Waltham, MA, USA) and 10% fetal bovine serum (FBS) (Corning, Inc., Corning, NY, USA) in a 5% CO_2_, humidified atmosphere at 37 °C, according to the American Type Culture Collection directory’s instructions.

### 4.2. Cultivation of Human Umbilical Cord Vein Endothelial Cells 

Human umbilical cord vein endothelial cells were purchased from the American Type Culture Collection. HUVECs were cultured in Medium 199 (M199; Thermo Fisher Scientific), supplemented with 10% fetal bovine serum (Corning), 10 U/mL heparin (Sigma-Aldrich), 20 μg/mL endothelial cell growth supplement (ECGS; BD Biosciences, San Jose, CA, USA), and 1% penicillin/streptomycin (P/S) (Life Technologies, Carlsbad, CA, USA) in a 5% CO2, humidified atmosphere at 37 °C, as described in our previous report [33]. Culture media were changed every other day and we subcultured the cells when the HUVEC culture reached 80% confluency. 

### 4.3. Assessment of MicroRNA Expression by Quantitative RT-PCR 

Total microRNA in cultured cervical cancer cell lines was isolated using MicroRNA isolation kits (BioChain Institute, Inc., Hayward, CA, USA) according to the manufacturer’s instructions [63]. Aliquots of total microRNA (equivalent to 100 ng total RNA) were combined with a reverse transcription (RT) mixture (Ambion, Inc., Austin, TX, USA) and reverse-transcribed into complementary deoxyribonucleic acid (cDNA). All of the templates were mixed with polymerase chain reaction (PCR) mixtures and double that amount of TaqMan^®^ Universal PCR Master Mixture, and then we performed a PCR amplification with an ABI 7900 Detection System (Applied Biosystems, Foster City, CA, USA) according to the manufacturer’s instructions. Specific RT and PCR primers for endogenous control housekeeping gene *U6* small nuclear RNA (U6) or *miR-128* were purchased from Ambion, Inc. The fold change was estimated as 2−ΔΔ*C*t, where ΔΔ*C*t = Δ*C*ttreatment − ΔCtsham control and Δ*C*t = Cttarget gene − CtU6, as we previously reported [63].

### 4.4. Transfection of MicroRNA-128 mimic and MicroRNA-128 Inhibitor 

Synthetic *miR-128* mimic oligonucleotides, *miR-128* inhibitor oligonucleotides, and scrambled controls were obtained from GenePharma, Inc. (Shanghai, China). Cervical cancer cells were incubated until subconfluency and transfected with *miR-128* mimic, *miR-128* inhibitor, or a scrambled control by using Lipofectamine RNAiMax transfection reagent (Life Technologies) in Opti-MEM I Reduced Serum Medium (Life Technologies) at a final concentration of 10 or 30 nM (*miR-128 mimic*) or 10- or 100 nM (*miR-128 inhibitor*), according to the manufacturer’s instructions [64]. 

### 4.5. Parallel-Plate Flow Chamber Culture System

HUVECs were obtained and cultured as described in Section 4.2. The first 2‒3 passages of HUVECs were trypsinized and seeded onto a 24 mm × 50 mm cover glass slide. A parallel-plate flow chamber assembled with a glass slide was suffused for 24 h with a medium for HUVECs, as in our previous report [33,42], and the shear flow force was generated and controlled using an oscillatory peristaltic pump (Instech Laboratories, Inc., Plymouth Meeting, PA, USA) to generate various shear forces through the chamber. 1 × 105 GFP-expressed cervical cancer cells were mixed with the medium and perfused through the flow chamber for 48 h at 1 dyn/cm^2^, 2.5 dyn/cm^2^, and 5 dyn/cm^2^ shear flow rates. Three independent experiments were performed using different batches of cells.

### 4.6. Isolation of Shear Stress-Resistant Cervical Cancer Cells

Studies have demonstrated that circulating cancer cells moving toward a distant metastasis are very rare in the bloodstream [40]. We adjusted the shear forces to decrease the viability of cervical cancer cells to less than 10% (defined as a shear stress-resistant clone, as previously described [8]). Viable GFP-expressed cervical cancer cells that adhered to the HUVEC layer and were resistant to shear stress in the parallel-plate flow chamber were further trypsinized using a real-time fluorescence videoing system under sterile conditions. GFP-expressed WT/SSR cervical cancer cells were harvested and sorted according to the manufacturer’s instructions [65]. In brief, GFP-expressed WT/SSR cervical cancer cells adhering on HUVECs in a flow chamber were harvested using 0.05% Trypsin/EDTA (Gibco, Grand Island, NY, USA). A trypsinized cell suspension may contain HUVECs and GFP-expressed cervical cancer cells; therefore, we sorted GFP-expressed cervical cancer cell populations using the cell sorter FACSAria™ III (BD Biosciences) on the cell mixture to separate them from HUVEC endothelial cells. Then, the isolated GFP-cervical cancer cells were incubated in a basal optimal growth medium as described in Section 4.1, and further cultured for at least three passages. Immunostaining GFP fluorescence and selective HPV 16/18 E6 oncoprotein markers were used to confirm that the isolated GFP-expressed cell clones were indeed cervical cancer cells without contamination from HUVEC endothelial cells. For the mechanistic study of SSR cervical cancer cells, clones of interest were maintained in the basal medium until confluence. For parental WT cervical cancer cells, viable cervical cancer cells adhering to the HUVEC layer without exposure to shear force in the chamber were isolated. Each cell clone was repeatedly selected by the flow chamber system at least three times. 

### 4.7. Assessment of Static Cell Adhesion Capacity of SSR and WT Cervical Cancer Cells by Flow Chamber Adhesive Assay 

We wanted to verify the static adhesion capacity of SSR and WT cells and evaluate the effects of *miR-128* on SSR/WT cell-mediated cell adhesion by using a modified flow chamber adhesive assay, as previously described [66]. Briefly, the glass slide plate of the flow chamber was precoated with 20 µg/mL laminin for 16 h and then we inoculated a monolayer of HUVECs on the coated surface. Various isolated WT and SSR clones of two cervical cancer cell lines were transected with synthetic miR-128 inhibitor oligonucleotides, *miR-128* mimic oligonucleotides, or scramble negative control oligonucleotides for 48 h. Later, we prelabeled SSR and WT cells (with or without *miR-128* inhibitor, *miR-128* mimic, or scramble negative control) with a fluorescent dye, as described in [67]. Briefly, 1 × 10^7^ cells/mL were incubated for 30 min at 37 °C in Opti-MEM (a reduced-serum medium) containing 10 µM of Cell TrackerTM fluorescent dye Orange Red CMRA (for SSR clones) or Blue CMF2HC (for WT clones). After washing, cells were incubated for an additional 30 min with dye-free PBS, washed, and harvested for the following experiments. Subsequently, mixtures containing 1 × 10^5^ CMRA prelabeled SSR cells and 1 × 10^5^ CMF2HC prelabeled WT cells were gently infused into the flow chamber and kept there for 12 min to allow the cervical cancer cells to settle onto the ECs. This was followed by perfusion with 0, 1, 2.5, or 5 dyn/cm^2^ shear for 10 min. The chamber was then inverted to allow the cells unattached to the ECs to settle at the bottom. After the EC layer was fixed with 1% glutaraldehyde, the number of SSR and WT cells that adhered to the EC layer was counted under a fluorescence microscope. Cells positive for blue (WT cells) or red (SSR cells) fluorescence reactions on the HUVECs layer were counted. The adhesion capacity of SSR cells was expressed as the number of cells positive for red or blue fluorescence. The experiment was repeated three times and at least six fields were counted per experiment.

### 4.8. Time-Lapse Recording of Cell Movement Assay 

Time-lapse recording of cell migration was performed according to the manufacturer’s instructions, as previously described [68]. Briefly, 1 × 10^5^/well (six-well plates) of various SSR or WT cervical cancer cells were transected with or without synthetic *miR-128* inhibitor oligonucleotides or *miR-128* mimic oligonucleotides for 48 h and then incubated in the basal medium, as described in Section 4.4. Phase-contrast images of time-lapse recordings for cell location were collected hourly using a microscope and CCD system (Axiovert 200; Carl Zeiss MicroImaging GmbH; Jena, Germany) over the next 8 h. Images were subjected to tracing semi-automatically after every single cell migration and movement (in µm) using AxioVision software. The migration speed was defined as the single cell movement (in µm)/8 h. 

### 4.9. Wound-Healing Assay

Time-lapse recording of cell migration was performed as previously described [62,68]. Various WT and SSR clones from two cervical cancer cell lines were transected with or without synthetic *miR-128* inhibitor oligonucleotides or *miR-128* mimic oligonucleotides for 48 h. Then, cells were incubated until the formation of a confluent monolayer in the absence of serum for 24 h and streaked out to a 300- to 400-μm width strip of cells across the well using a 10-µL pipette tip. The streaked regions were incubated in serum-free medium at 37 °C for 8 h. Wound-healing capacity was expressed as the percentage of the cell-free area of the initial streaked region/cell-free areas of the streaked region after an 8-h incubation using the public domain software ImageJ (http://rsbweb.nih.gov/ij/index.html). The experiment was repeated three times and at least six fields were counted per experiment.

### 4.10. Reverse Transcription and Quantitative PCR 

Procedures for RNA isolation, concentration, quality determination, RT-PCR, and quantitative PCR have been described previously [69]. Briefly, RNA was extracted using TRI Reagent (Sigma-Aldrich), treated with DNase I (Ambion), and reverse-transcribed with random primers (Invitrogen). A negative control that omitted the reverse transcriptase was always performed to ensure that the mRNA samples were not contaminated with genomic DNA. The primers used are listed in Table 1.

### 4.11. Statistical Analyses 

Each set of data was shown as the mean ± SD and was evaluated using the one-way ANOVA module of Prism 4.02 software (GraphPad Software, San Diego, CA, USA). The F-test and Tukey’s tests were used to assess whether the differences between the experimental results for paired groups were significant. Dunnett’s test was used to compare the results for multiple groups after the significance was found. Student’s *t*-test was utilized when two samples were compared. 

## Figures and Tables

**Table 1:** Primer list *.

Gene	NCBI Ref. No	Primer	Sequence
Human *ITGA5*	NM_002205	Forward	GCCGATTCACATCGCTCTCAAC
		Reverse	GTCTTCTCCACAGTCCAGCAAG
Human *ITGB5*	NM_002213	Forward	GCCTTTCTGTGAGTGCGACAAC
		Reverse	CCGATGTAACCTGCATGGCACT
Human *sLex*	NM_002033	Forward	GGGTTTGGATGAACTTCGAGTCG
		Reverse	GGTAGCCATAAGGCACAAAGACG
Human *18S rRNA*	NR_003286	Forward	GTGTGCCTACCCTACG
		Reverse	TGACCCGCACTTACTC
Human *CEACAM6*	NM_008084	Forward	GCCTCAATAGGACCACAGTCAC
		Reverse	AGGGCTGCTATATCAGAGCGAC
Human *MMP9*	NM_004994	Forward	GCCACTACTGTGCCTTTGAGTC
		Reverse	CCCTCAGAGAATCGCCAGTACT
Human *MMP23*	NM_006983	Forward	CACTTCGACGACAGCGAGTACT
		Reverse	GCCGTGTTGTGAGTGCATCAGG

* All primers were designed for both conventional and quantitative PCR.

## Supplementary Materials

The following are available online at https://www.mdpi.com/1422-0067/22/1/215/s1, Figure S1: A schematic diagram of the parallel-plate flow chamber and working flowchart, Figure S2: Validation of *miRNA-128* level in WT or SSR cervical cancer cells after transfection of scramble negative control, *miR-128 inhibitor*, or *miR-128 mimic* oligonucleotides, Figure S3: Investigation of the effects of knockdown of integrin 5 (ITGA5) or integrin 5 (ITGB5) on the static adhesion capacities in human cervical cancer cells, and evaluation of the impact of *miR-128* on ITGA5 or ITGB5 mediated on the adhesive ability between cervical cancer cells and endothelial cells.
