# Supplementary material for: MicroRNA-128 Confers Anti-Endothelial Adhesion and Anti-Migration Properties to Counteract Highly Metastatic Cervical Cancer Cells’ Migration in a Parallel-Plate Flow Chamber
Source: Int J Mol Sci. 2020 Dec 28;22(1):215. doi: 10.3390/ijms22010215 (PMC7796002; doi:10.3390/ijms22010215)
Supplement: Supplementary file 1 [file ijms-22-00215-s001.pdf]

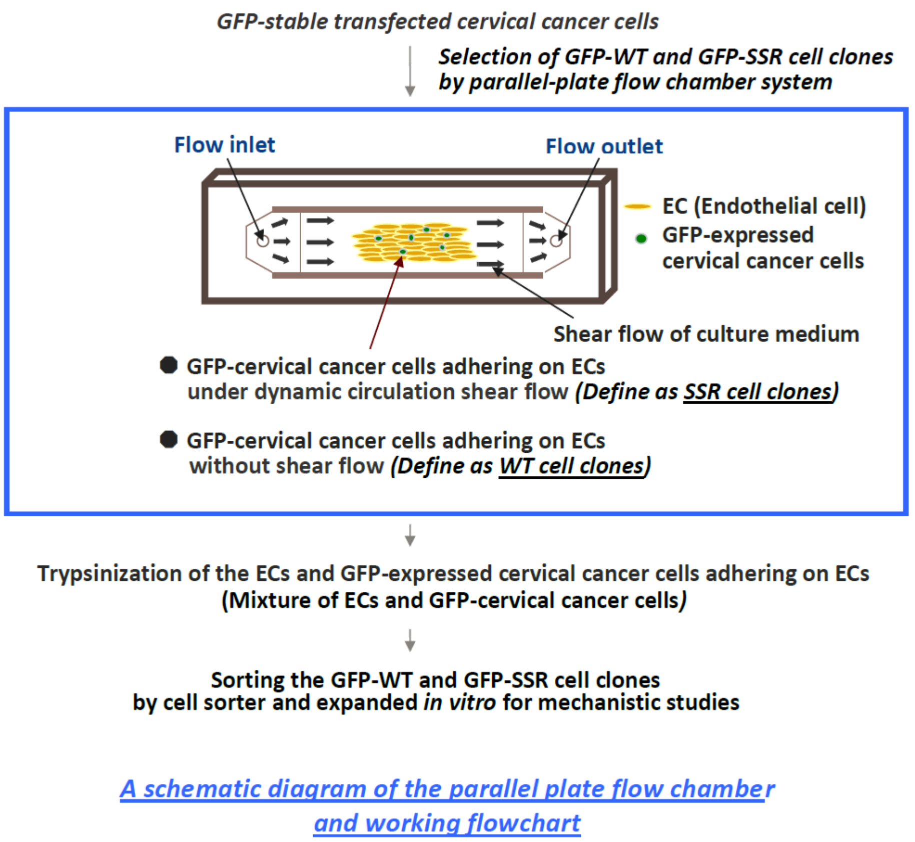

Figure S1. A schematic diagram of the parallel-plate flow chamber and working flowchart.

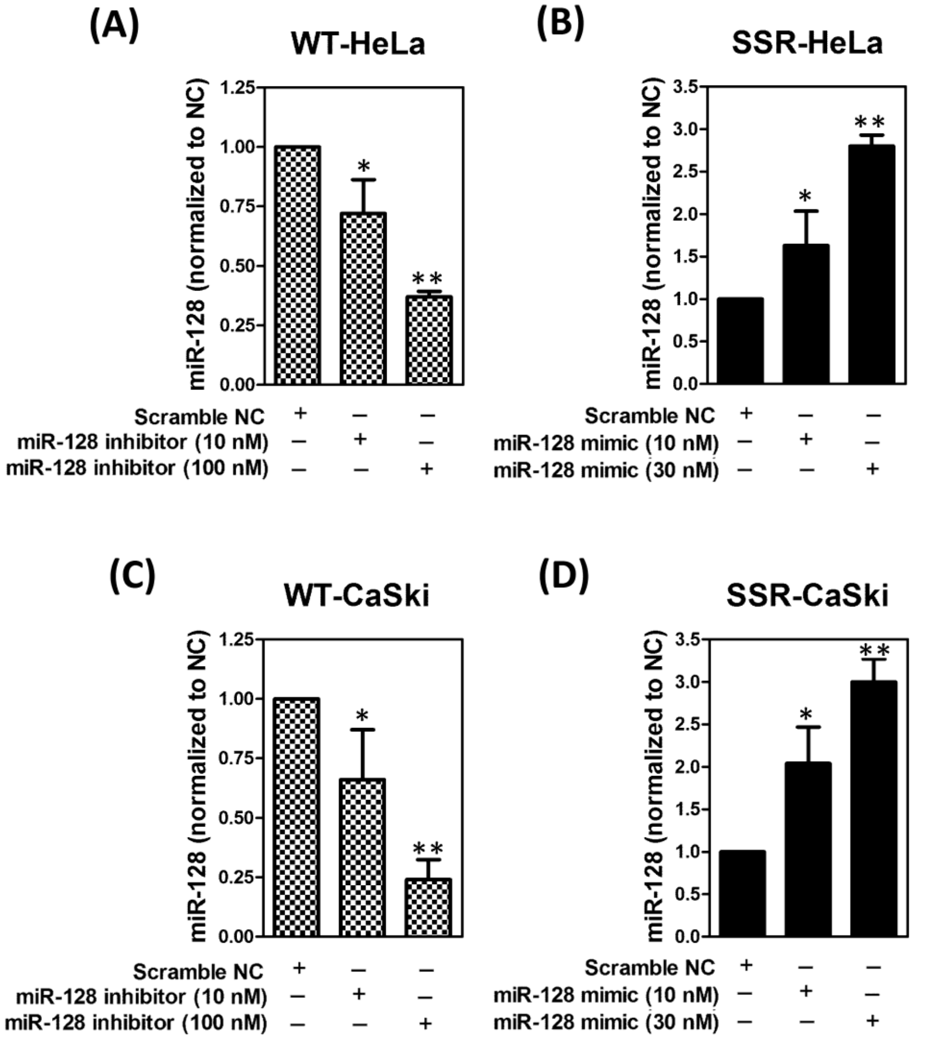

Figure S2. Validation of *miRNA-128* level in WT or SSR cervical cancer cells after transfection of scramble negative control, *miR-128 inhibitor*, or *miR-128 mimic* oligonucleotides. Quantitative RT-PCR analysis confirmed that (**A**) the *miR-128 inhibitor* transfection group dose-dependently reduced the *miR-128* transcripts in WT-HeLa cells compared to the scramble NC group. In contrast, (**B**) the *miR-12 8mimic* transfection group markedly enhanced the *miR-128* transcripts in SSR-HeLa cells compared to the scramble NC group. A similar phenomenon was seen in (**C**) the *miR-128 inhibitor* transfection group of WT-CaSki cells and (**D**) the *miR-128 mimic* transfection group of SSR-CaSki cells. Data show the mean and standard deviation (SD) of three independent experiments using different batches of cells. *$P < 0.05$; **$P < 0.01$ for the *miR-128 inhibitor* group vs. scramble NC group or *miR-128 mimic* group vs. scramble NC group.

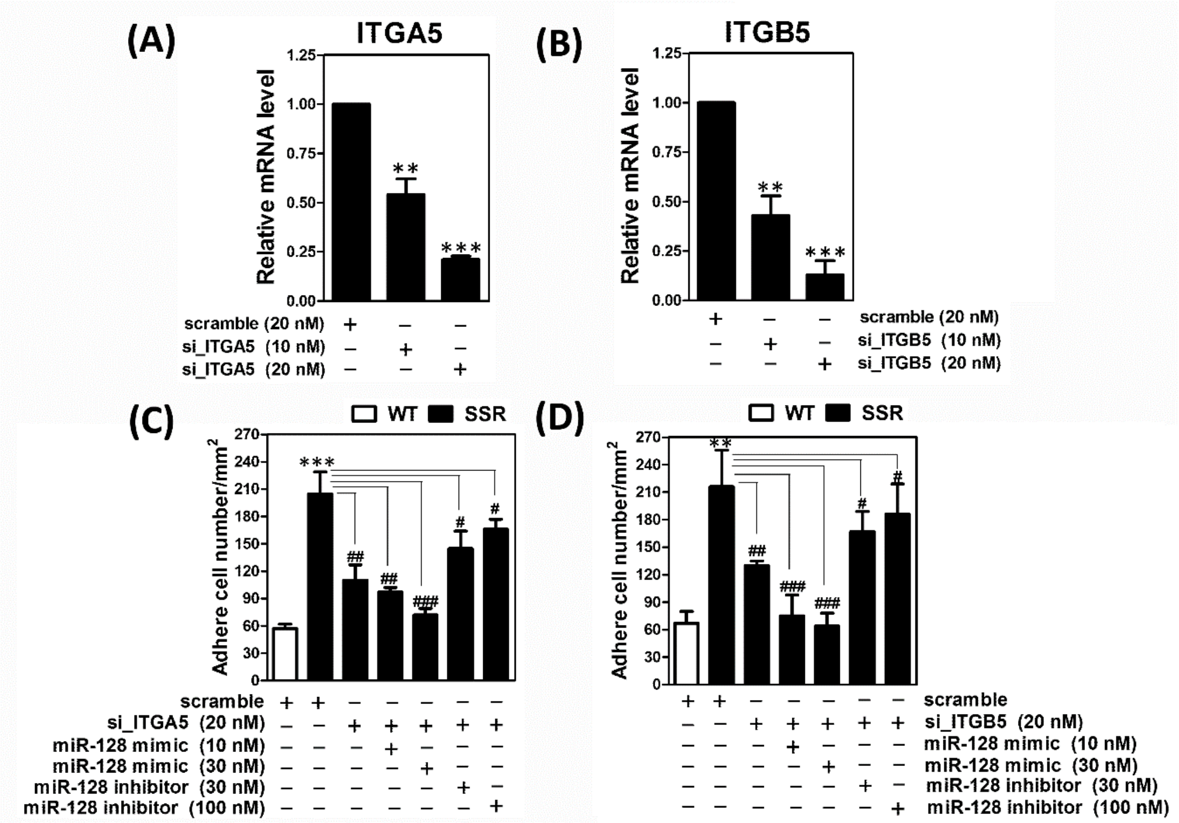

Figure S3. Investigation of the effects of knockdown of integrin α5 (ITGA5) or integrin β5 (ITGB5) on the static adhesion capacities in human cervical cancer cells, and evaluation of the impact of *miR-128* on ITGA5 or ITGB5 mediated on the adhesive ability between cervical cancer cells and endothelial cells. SSR-HeLa cells were transient transfected with scramble negative control oligonucleotides (scramble NC) or siRNA of **(A)** ITGA5 or **(B)** ITGB5 for 48 hours and the cell extracts were collected and estimated the transcript levels of ITGA5 or ITGB5. Data showed mean and standard deviation (SD) of three independent experiments using different batches of cells. $^{**}P < 0.01$, $^{***}P < 0.001$ for the si_ITGA5 group or si_ITGB5 group vs. scramble NC group. HeLa cells were pre-transfected with si-RNA of **(C)** ITGA5 or **(D)** ITGB5, and then were exogenously introduced of synthetic *miR-128* inhibitor oligonucleotides, *miR-128* mimic oligonucleotides or scramble negative control oligonucleotides for another 48 hours and then performed the static adhesion assay in human WT or SSR-HeLa cells. All the data were repeated for three times and at least six fields per individual experiment were counted. Each data set was presented as the mean ± SD; $^{**}P < 0.01$, $^{***}P < 0.001$ for the SSR scramble group vs. WT scramble NC group. $^{\#}P < 0.05$, $^{\#\#}P < 0.01$, $^{\#\#\#}P < 0.001$ for the SSR+siITGA5 or SSR+siITGB5 group vs. SSR+*siITGA5* or SSR+*siITGB5 +miR-128 mimic* group, or the SSR+*siITGA5* or SSR+*siITGB5* group vs. SSR+*siITGA5* or SSR+*siITGB5 +miR-128 inhibitor* group.
